# Disparity in peripheral and renal B-cell depletion with rituximab in systemic lupus erythematosus: an opportunity for obinutuzumab?

**DOI:** 10.1093/rheumatology/keab827

**Published:** 2021-11-11

**Authors:** Venkat R Reddy, Ruth J Pepper, Kavina Shah, Geraldine Cambridge, Scott R Henderson, Christian Klein, Loren Kell, Samuel J Taylor, David A Isenberg, Mark S Cragg, Maria J Leandro

**Affiliations:** Department of Rheumatology, University College London Hospitals NHS Foundation Trust; Centre for Rheumatology and Bloomsbury Rheumatology Unit, University College London; Department of Rheumatology, University College London Hospitals NHS Foundation Trust; Department of Renal Medicine (formerly Centre for Nephrology), Royal Free Hospital, London, UK; Department of Rheumatology, University College London Hospitals NHS Foundation Trust; Centre for Rheumatology and Bloomsbury Rheumatology Unit, University College London; Centre for Rheumatology and Bloomsbury Rheumatology Unit, University College London; Department of Renal Medicine (formerly Centre for Nephrology), Royal Free Hospital, London, UK; Cancer Immunotherapy Discovery, Oncology Discovery & Translational AreaRoche Pharma Research & Early Development, Roche Innovation Center, Zurich, Switzerland; Centre for Rheumatology and Bloomsbury Rheumatology Unit, University College London; Centre for Rheumatology and Bloomsbury Rheumatology Unit, University College London; Department of Rheumatology, University College London Hospitals NHS Foundation Trust; Centre for Rheumatology and Bloomsbury Rheumatology Unit, University College London; Centre for Cancer Immunology, Faculty of Medicine, University of Southampton, Southampton, UK; Department of Rheumatology, University College London Hospitals NHS Foundation Trust; Centre for Rheumatology and Bloomsbury Rheumatology Unit, University College London

**Keywords:** B cell depletion, rituximab, SLE, LN, obinutuzumab

## Abstract

**Objectives:**

To investigate key factors that may contribute to the variability of rituximab-mediated peripheral and renal B cell depletion (BCD) in SLE.

**Methods:**

We analysed: (i) CD19+ B cell counts in patients with SLE before and 1, 2, 3 and 6 months after treatment with rituximab, comparing them with RA patients; (ii) the presence of B cells in renal biopsies after rituximab therapy; (iii) whether the duration of BCD correlated with patient demographics and B cell expression of CD20 and FcγRIIb; and (iv) the effect of B cell activation factor (BAFF) on the efficiency of rituximab and obinutuzumab at inducing BCD in whole blood assays, *in vitro*.

**Results:**

In SLE (*n* = 71), the duration of BCD was shorter compared with RA (*n* = 27). B cells were detectable in renal biopsy samples (*n* = 6) after treatment with rituximab in all patients with poor response while peripheral blood B cells remained low or undetectable in the same patients. There were no significant relationships between peripheral BCD and patient age, disease duration, serum C3 levels or the level of expression of B cell surface proteins CD20 and FcγRIIb. Obinutuzumab was more efficient than rituximab at inducing BCD in whole blood assays, regardless of excess BAFF.

**Conclusions:**

BCD in SLE is less efficient than in RA. Renal B cell presence following rituximab treatment was associated with poor outcomes. No significant relationships between any measured B cell related, clinical or laboratory parameters and the efficiency of BCD by rituximab was found. Obinutuzumab was superior to rituximab at inducing BCD.

Rheumatology key messages

- B cell depletion in SLE is less efficient than RA regardless of clinical and laboratory parameters.
- Disparity in peripheral and renal B-cell depletion is notable in rituximab refractory LN.
- Obinutuzumab was superior to rituximab at inducing B cell depletion *in vitro*, regardless of B cell activation factor.

## Introduction

In patients with SLE, incomplete peripheral blood B cell depletion (BCD) contributes to poor response to therapy using the anti-CD20 mAb, rituximab [1, 2]. The presence of renal interstitial B cells in patients with LN is associated with renal dysfunction and histologically active lesions [3]. Rituximab treatment for refractory LN results in variable peripheral BCD [4] and the lupus nephritis assessment with rituximab (LUNAR) study found that complete peripheral BCD (0 cells/µl) lasting >71 days was associated with complete response [4, 5]. In a study of seven patients with LN who responded well to rituximab, BCD in the kidneys was reported in all patients on repeat renal biopsy at 3–12 months after rituximab [6]. Therefore, improving BCD may enhance clinical response [7, 8]. However, we do not know whether peripheral blood BCD reflects BCD in the kidney and if incomplete BCD in the kidney fails to disrupt inflammatory lesions, contributing to poor response.

Anti-CD20 mAbs can employ multiple effector mechanisms including complement-dependent cell cytotoxicity (CDC), antibody-dependent cellular cytotoxicity, antibody-dependent cell phagocytosis and direct cell death [9]. There are two main types of anti-CD20 mAbs: Type I anti-CD20 mAbs cluster the target B cell antigen, CD20, and recruit the inhibitory FcγRIIb, CD32b, leading to their internalization to a significantly greater extent than Type II anti-CD20 mAbs such as obinutuzumab [10–12]. Clustering of anti-CD20 mAbs facilitates efficient CDC, whereas internalization impairs other Fc-mediated effector functions such as antibody-dependent cellular cytotoxicity [10] and antibody-dependent cell phagocytosis.

In SLE, both inherent and acquired defects in effector mechanisms compromise the efficiency of anti-CD20 mAbs. We have previously shown that B cell intrinsic mechanisms such as B cell internalization of rituximab [13] and B cell extrinsic factors such as rituximab pharmacokinetics [14] influence its efficiency. SLE-related defects in complement system [15], phagocytosis [16] and elevated serum B cell activation factor (BAFF) levels [17] may also limit the efficiency of rituximab in SLE [17–20]. BAFF antagonizes deletion of self-reactive B cells in transgenic mice [21], and promotes anti-apoptotic protein expression on B cells, which may compromise rituximab-induced apoptotic direct cell death [10]. In SS, BAFF was reported to modulate repopulation of B cells in patients treated with rituximab [22]. However, there are no current data comparing the duration of BCD in SLE with other autoimmune diseases without such disease-related defects in the complement system or phagocytosis such as RA, where the efficacy is more predictable [23]. Furthermore, we do not know whether peripheral blood BCD reflects BCD in the kidney and data about the relationship between renal BCD and clinical response to rituximab is limited. Therefore, it is important to understand whether host- and/or disease-related factors influence the efficiency of anti-CD20 mAb-mediated BCD in SLE.

In SLE, host-specific factors such as FcγRIIIa polymorphisms are reported to influence the efficiency of rituximab [19], but the effect of patient demographics is not known. Given SLE-related complement defects [18], it is also important to probe the relationship between serum C3 levels and the efficiency of BCD. Furthermore, the relationship between B cell intrinsic factors such as expression of the target antigen, CD20, and FcγRIIb (CD32b) that mediates internalization of rituximab, and extrinsic factors such as the effect of excess BAFF on the efficiency of anti-CD20 mAbs is not known.

Following on from our previous studies [10, 13, 14], here we compared the duration of BCD in patients with SLE and RA, analysed BCD in the kidney samples from patients with refractory LN with poor response to rituximab and explored the relationship between possible factors influencing the efficiency of rituximab, *in vivo* and *in vitro*. A recent phase II study reported good clinical efficacy of obinutuzumab in proliferative LN [24]. Therefore, we investigated whether obinutuzumab, given its superiority in inducing BCD *in vitro* [10], was capable of delivering superior BCD in the presence of excess BAFF.

## Methods

### Patients

This was a retrospective study. All patients participating in this study fulfilled the ACR (ACR/SLICC) classification criteria for SLE or (ACR/EULAR) diagnostic criteria for RA, respectively. All study participants provided written informed consent according to the declaration of Helsinki and in the remit of the London-Bentham Research Ethics Committee approval of the study. All patients were treated with rituximab (1 g × 2, given 2 weeks apart) at University College Hospital, London, UK. Patient demographics, and clinical and laboratory parameters of the cohort have been described previously [10, 13, 25]. Rituximab was a gift from the pharmacy of University College Hospital. Roche Innovation Center Zurich, Switzerland provided obinutuzumab [26, 27].

### Flow cytometry

Fluorochrome-conjugated mAbs anti-CD3 [phycoerythrin (PE)-Cy7], anti-CD19 (Alexa Fluor 700), anti-CD45 (PE), anti-CD20 (FITC) and anti-CD32 (PE) were obtained from BD Biosciences (Oxford, UK) and Biolegend, London, UK. In addition to forward- and side-scatter characteristics, B cells were identified as CD19+ and T cells as CD3+, by flow cytometry using a Becton Dickinson LSR Fortessa cell analyser (supplementary Figs S1 and S2, available at *Rheumatology* online). Peripheral blood mononuclear cells were separated from whole blood by Ficoll-Hypaque density gradient and B cells were isolated using EasySep Human B Cell Enrichment Kit (Stemcell Technologies, Cambridge, UK).

### Whole blood BCD assays

Whole blood BCD assays were performed as described previously [10]. Briefly, freshly drawn, heparinized whole blood (100 µl) was incubated with or without mAbs at 1 µg/ml for 24 h at 37°C and 5% CO_2_ and subsequently analysed by flow cytometry. Samples were incubated with BAFF at 100 ng/ml for some experiments. The cytotoxicity index (CTI) of mAbs was calculated from the proportion of B cells to T cells on flow cytometry, as described previously [13].

### Immunohistochemistry on renal biopsies

Renal biopsy was performed in six patients with suspected active LN despite previous or ongoing treatment with immunosuppression and following treatment with rituximab (1 g × 2, given 2 weeks apart). All patients had a renal biopsy between 5 months and 11 years after rituximab treatment. Immunohistochemistry was performed on formalin-fixed paraffin-embedded sections using standard techniques to stain for B cells using 2 different antibodies: anti-PAX-5 (Becton Dickinson, UK) and anti-CD79 (Dako, Agilent Technologies LDA UK Limited) at a dilution of 1:200 and incubated for 1 h at room temperature. Antigen retrieval was performed using citrate buffer. Following the addition of a secondary antibody and a washing step, Avidin-Biotin Complex solution was added. After a further washing step, the slides were developed using 3,3-diaminobenzidine.

### Statistical analysis

Statistical analysis was performed using Graphpad Prism software (version 5). Mann–Whitney *U* test or paired *t*-tests were used to compare unpaired or paired samples, respectively. Spearman rank sum test (r^2^) was used to analyse relationships between variables and r^2^ > 0.45 and *P* < 0.05 were considered significant.

## Results

### Duration of BCD in RA and SLE

**Fig. 1:**
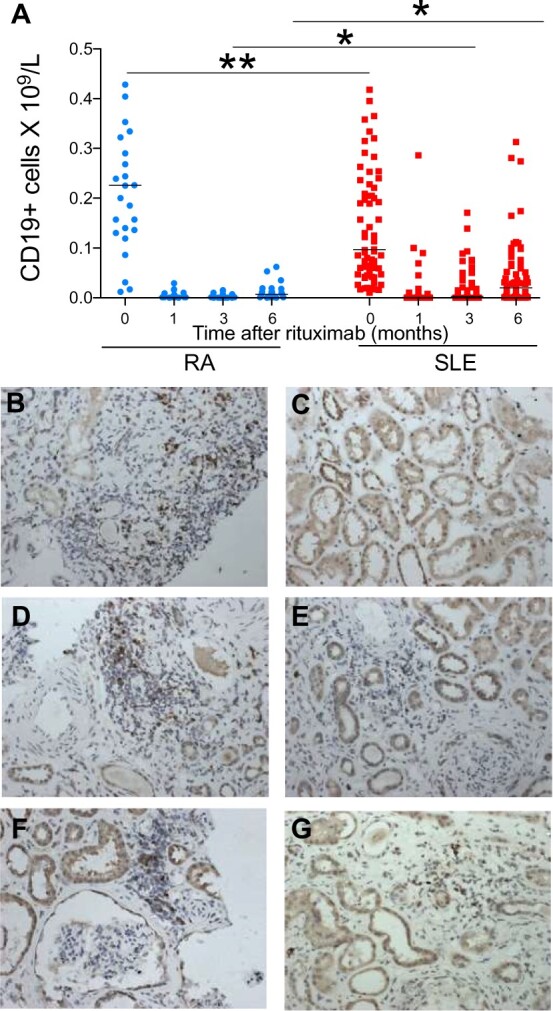
B cell depletion following rituximab (**A**) Peripheral and renal B cell depletion with rituximab in patients with RA and SLE. CD19+ B cell counts in peripheral blood before and after treatment with rituximab in patients with RA (*n* = 27) and SLE (*n* = 71). (**B**–**G**) Immunohistochemistry photomicrographs ×200 of anti-CD79 staining in renal biopsies, which illustrates the degree of B cell infiltration in the biopsies from patients 1–6, respectively. *P < 0.05; **P < 0.001.

The duration of BCD was assessed in 27 patients with RA and 71 patients with SLE. Before treatment with rituximab, patients with RA had significantly higher number of CD19+ cells (median 0.2260 × 10^9^/l, range 0.012–0.663) in peripheral circulation, compared with patients with SLE (median 0.097 × 10^9^/l, range 0.01–1.274) (Fig.  1A) (Mann–Whitney *U* test). In contrast, at both 3 and 6 months after treatment with rituximab, the number of CD19+ cells was significantly lower in patients with RA compared with patients with SLE. No significant differences were noted at 1 month after rituximab, although some patients with SLE had CD19+ cell counts >0.5 × 10^9^/l. These results showed that after rituximab treatment the duration of BCD was shorter in patients with SLE compared with patients with RA.

### BCD in the kidney

A repeat renal biopsy was performed in patients with suspected active LN for the following indications: decline in renal function in four patients (with nephrotic-range proteinuria in three patients) and nephrotic syndrome with preserved renal function in the remaining two patients. Renal biopsy was performed at a median of 12 weeks (range 2–15 weeks’ time from second or only dose of rituximab).

At the time of the renal biopsy following rituximab, the median creatinine was 375 μmol/l (range 54–764), median urine protein creatinine ratio was 632 mg/mmol (range 85–969) and median albumin was 26 g/l (range 18–32). Table  1 demonstrates the individual results and class of LN on both renal biopsies as well as treatment details.

**Table 1:** Renal biopsy data and parameters before and after rituximab

Patient	Prior-renal biopsy class after RTX	Previous treatment	Duration between biopsies	Class of renal biopsy after RTX	Treatment at time of biopsy	Creatinine (μmol/l)	PCR (mg/mmol)
1	IV + V	CS, CYC, MMF, AZA, CSA	7 years	V	CS, MMF	66	371
2	N/A	CS, AZA, MMF, CSA	N/A	V	CS, MMF	54	637
3	IV	CS, CYC	11 years	IV-GA/C	CS	764	969
4	IV G/A	CS, CYC, MMF	8 months	VI	CS, CYC	394	883
5	IV + V	CS, MMF	5 months	IV	CS, MMF	461	85
6	IV-G + V	CS, CYC	5 months	IV-GA/C, V	CS, ×1 dose CYC	356	627

AZA: azathioprine; CS: corticosteroids; CYC: cyclophosphamide; MMF: mycophenolate mofetil; PCR: protein–creatinine ratio; N/A: not available; RTX: rituximab.

During this episode of active disease at the time of the biopsy, three patients had elevated anti-dsDNA antibody levels of 1766, 76 and 124 IU/ml, and three other patients had normal levels (10, 15 and 19 IU/ml). Of these patients with normal anti-dsDNA antibody levels, two had higher levels prior to treatment with rituximab. Five patients had low serum C3 levels (<0.9 g/l).

Patients 2 and 4 had minimal staining with anti-CD79. The remaining four patients had detectable B cells demonstrated by anti-CD79 staining (Fig.  1B–G). Immunohistochemistry with anti-Pax-5 showed similar staining patterns to that with anti-CD79 in four patients (Fig.  1C). Two patients without detectable renal B cells on both staining patterns had Class V and Class VI LN; therefore, staining was only present in proliferative glomerular lesions.

### Peripheral B cells in patients with LN

Complete BCD was defined as ≤ 0.005 × 10^9^/l. Patient 2 with a Class V lesion and normal renal function at follow-up had no detectable B cell infiltration, despite a CD19 count of 0.03 × 10^9^/l at the time of renal biopsy. This patient had briefly reached a nadir CD19 count of 0.006 but reconstituted rapidly without detectable renal B cell infiltration. In this cohort of patients, patient 2 is the only patient with normal renal function at long-term follow-up. Patient 1 also with a Class V lesion and a CD19 count of 0.042 × 10^9^/l demonstrated renal B cell infiltration. Patients 3, 5 and 6 had significantly suppressed CD19 count (≤0.002) with all three patients having detectable anti-CD79 staining in renal biopsy samples. Patients 1, 3, 5 and 6 either progressed rapidly to end-stage renal failure or advanced chronic kidney disease (CKD). Table  2 demonstrates the correlation between renal biopsy B cells and peripheral B cells and their clinical outcomes. Patients with lower levels of renal B cells, irrespective of the peripheral B cell count, had better clinical outcomes.

**Table 2:** Peripheral CD19+ B cell counts and staining for B cells in renal biopsy samples and patient outcomes following rituximab treatment

Patient	Class of LN	Renal biopsy B cell staining		Peripheral B cells	Outcome at latest f/u
		Anti-CD79	Anti-Pax-5		
1	V	↑	↑	↑ 0.042 × 10^9^/l	Progressive CKD
2	V	→	→	↑ 0.03 × 10^9^/l	Remission (normal renal function)
3	IV-GA/C	↑	↑	↓ (≤0.002)	Dialysis → ESRF
4	VI	→	→	↓ (≤0.002)	Stable CKD (on PD)
5	IV	↑	↑	↓ (≤0.002)	Stable CKD
6	IV-GA/C	↑	↑	↓ (≤0.002)	Advanced CKD, dialysis dependent

→ minimal staining; ↑detectable B cells; ↓ suppressed counts (≤0.005). CKD: chronic kidney disease; ESRF: end stage renal failure; f/u: follow-up.

### B cell expression of CD20 and FcγRIIb in RA and SLE

Expression of CD20 and the inhibitory FcγRIIb on malignant B cells influences clinical response to rituximab in lymphoma [12, 28, 29]. Therefore, their expression on B cells from patients with RA (*n* = 44) and SLE (*n* = 81) were compared.

**Fig. 2:**
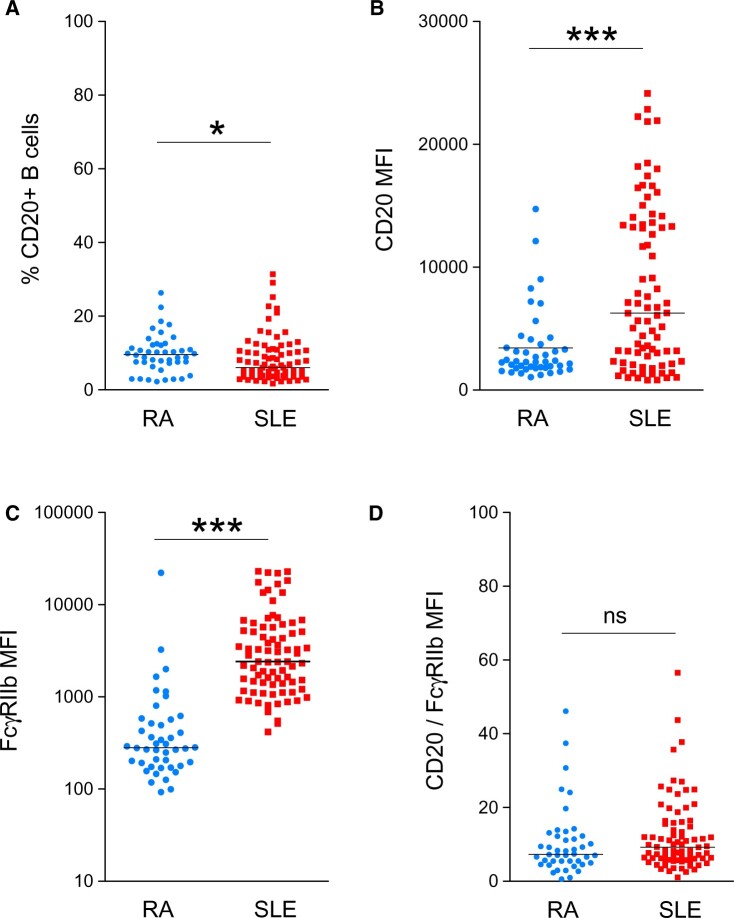
The MFI of CD20 and FcγRIIb on B cells from patients with RA and SLE (**A**) The frequency of CD20+ B cells in patients with RA and SLE; (**B**) the MFI of CD20 in patients with RA and SLE; (**C**) the MFI of FcγRIIb in patients with RA and SLE; and (**D**) the ratio of MFI of CD20 and MFI of FcγRIIb of CD20+ B cells from patients with RA and SLE. MFI, mean fluorescence intensity; *P < 0.05; ***P < 0.0001; ns: not significant.

The median [interquartile range (IQR)] frequency of CD20+ B cells was significantly greater in patients with RA compared with those with SLE, with 9.6% (7.3–12.1%) and 6% (3.9–10.8%), respectively (Fig.  2A). In contrast, B cell expression of CD20 [mean fluorescence intensity (MFI)] was significantly lower in patients with RA compared with those with SLE with median (IQR) 2292 (1787–3654) and 6261 (2334–13 446), respectively (Fig.  2B). However, the MFI of CD20 varied remarkably, particularly in patients with SLE.

B cell expression of FcγRIIb (CD32) (MFI) was also significantly lower in patients with RA compared with those with SLE, with median (IQR) 280 (181–555) and 2414 (1378–5707), respectively. The MFI of FcγRIIb varied remarkably between patients with RA and SLE (Fig.  2C). Further, there was no difference in the relative B cell expression of CD20 and FcγRIIb (ratio MFI of CD20: MFI of FcγRIIb) between patients with RA and SLE (Fig.  2D).

Thus, these results found no significant relationship between the relative expression of CD20 and FcγRIIb between patients with RA and SLE.

### Relationships between the efficiency of type I anti-CD20 mAb and patient demographics

**Fig. 3:**
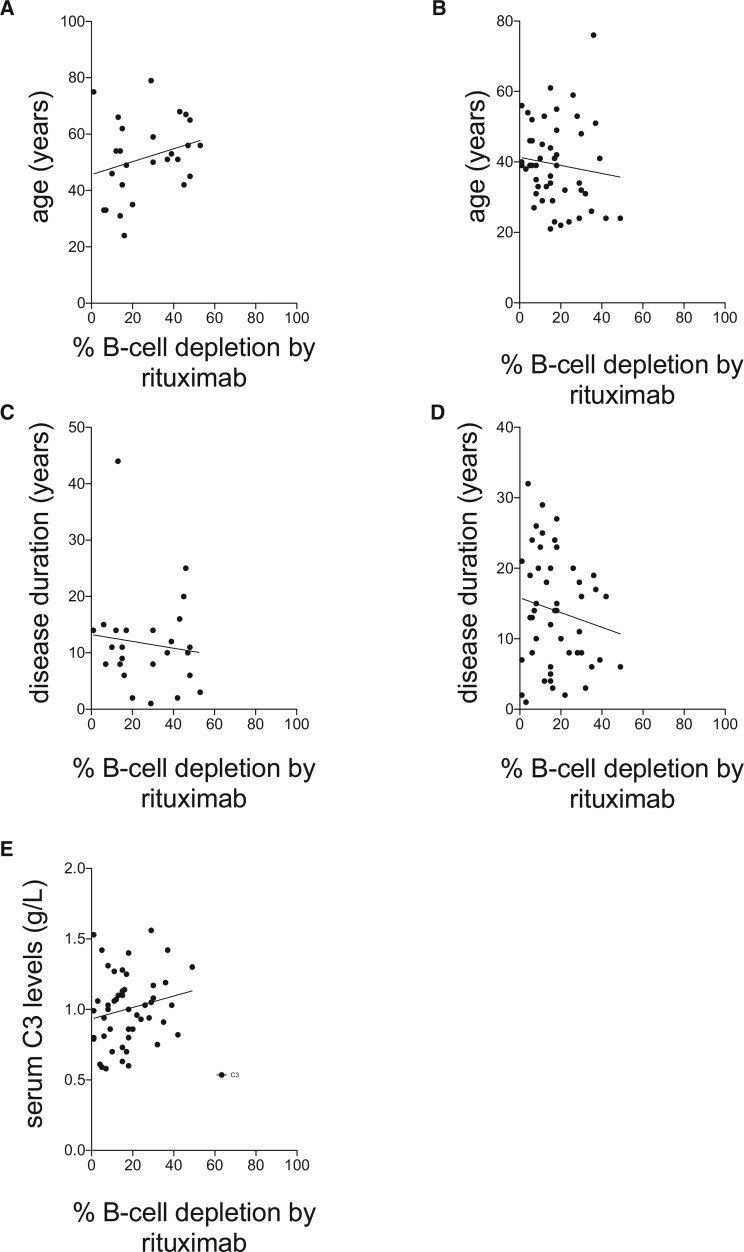
Relationships between patient demographics and BCD *in vitro* Relationships between rituximab-mediated BCD and age of patients with (**A**) RA and (**B**) SLE, and disease duration of patients with (**C**) RA and (**D**) SLE, and (**E**) serum C3 levels in patients with SLE. BCD: B cell depletion.

To assess the effects of patient characteristics on the efficiency of anti-CD20 mAbs, whole blood BCD assays were performed. There were no significant relationships between the percentages of BCD achieved by rituximab in the whole blood BCD assay in samples from patients with SLE and age (years) of patients with RA or SLE with r^2^, Spearman’s correlation coefficient, values of 0.07 (*P* > 0.1) and 0.01 (*P* > 0.4), respectively, or disease duration of patients with RA or SLE with r^2^, Spearman’s correlation coefficient, values of 0.01 (*P* > 0.5) and 0.02 (*P* > 0.2) or serum C3 levels with spearman r^2^ of –0.17 (*P* = 0.37), respectively (Fig.  3).

### BCD *in vitro* with type I and II anti-CD20 mAbs: relationship with B cell expression of CD20 and FcγRIIb

**Fig. 4:**
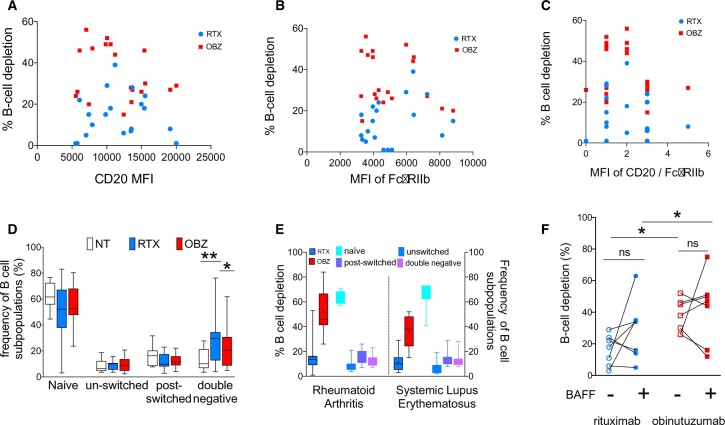
BCD *in vitro* with rituximab and obinutuzumab: relationship with B cell expression of CD20 and FcγRIIb; B cell subpopulations and the effect of BAFF Relationship between percentage BCD with rituximab and obinutuzumab in whole blood assays and (**A**) the MFI of CD20, (**B**) the MFI FcγRIIb and (**C**) the MFI of CD20/FcγRIIb on B cells from patients with SLE (*n* = 19). (**D**) Composition of B cell subpopulations in the whole blood BCD assay. The frequency of various B cell subpopulations in samples incubated with or without mAbs in samples from patients with RA (*n* = 9). (**E**) CTI of mAbs in whole blood BCD assay and the distribution of B cell subpopulations in RA (*n* = 10) and SLE (*n* = 9) in peripheral blood prior to incubation with mAbs. (**F**) The effect of excess BAFF on the efficiency of BCD. Each symbol represents an individual sample and the unfilled symbols represent samples incubated without excess BAFF and the filled symbols represent samples incubated with excess BAFF. Naïve, IgD+CD27–; unswitched memory B cells, IgD+CD27+; switched memory B cells, IgD–CD27+; double negative, IgD–CD27–. BAFF: B cell activation factor; BCD: B cell depletion; CTI: cytotoxicity index; MFI, mean fluorescence intensity; NT, not treated; RTX, rituximab; OBZ, obinutuzumab; *P < 0.05 and **P < 0.001; ns: not significant.

CD20 expression on B cells varied remarkably between patients with SLE with a mean (s.d.) of 11 555 (4354). However, there were no significant relationships between B cell expression of CD20 and the CTI of rituximab or obinutuzumab in the whole blood assay in samples from patients with SLE, with r^2^, Spearman’s correlation coefficient, values of –0.11 (*P* = 0.63) and 0.19 (*P* = 0.42), respectively (Fig.  4A).

Expression of FcγRIIb was less variable than that of CD20 with a mean (s.d.) of 5008 (1687). No significant correlations were found between the expression of CD20, FcγRIIb or their relative expression (ratio of MFI of CD20/FcγRIIb) to the CTI of rituximab or obinutuzumab in patients with SLE with r^2^, Spearman’s correlation coefficient, values of –0.31 (*P* = 0.19) and 0.25 (*P* = 0.29), respectively (Fig.  4A–C).

### BCD *in vitro* with type I and II anti-CD20 mAbs: relationship with the composition of B cell subpopulations

B cell subpopulations may differ in their susceptibility to deletion by mAbs [30]. Therefore, the distribution of B cell subpopulations in samples from nine patients with SLE incubated with anti-CD20 mAbs for 24 h in the whole blood BCD assay was analysed. There were no differences between B cell subpopulations in samples incubated with the two mAbs except for the frequency of double (CD19+IgD–CD27–) negative cells, which was higher in samples incubated with rituximab compared with samples incubated without mAbs and those incubated with obinutuzumab (Fig.  4D). The CTI of obinutuzumab was significantly greater than the CTI of rituximab in both RA (*n* = 10) and SLE (*n* = 9) (Fig.  4E). However, there were no significant differences in the frequencies of B cell subpopulations between the cohort of patients with RA and SLE (Fig.  4E). Collectively, these results suggest that obinutuzumab was more efficient at BCD than rituximab regardless of the composition of B cell subpopulations.

### Efficiency of type I and II anti-CD20 mAbs and the effect of excess BAFF

Patients with SLE may have high BAFF levels detectable in the serum [31]. Therefore, the effect of BAFF on the efficiency of anti-CD20 mAbs at inducing BCD was assessed by comparing the CTI of anti-CD20 mAbs in samples incubated in the presence or absence of BAFF at 100 ng/ml in the whole blood assay using freshly drawn blood samples from patients with RA (*n* = 3) and SLE (*n* = 4).

There were no consistent effects of excess BAFF on the efficiency of mAbs in the whole blood BCD assay in samples from patients with SLE (*n* = 7, Fig.  4F). There were no significant differences between the CTI of rituximab or obinutuzumab in the absence or presence of excess BAFF in the whole blood assay (*P* = 0.1875 and *P* > 0.05, respectively). The median (range) CTI of rituximab in the absence of excess BAFF was 18 (1–29) and in the presence of excess BAFF the median (range) CTI was 34 (14–63). The median (range) CTI of obinutuzumab in the absence of excess BAFF was 45 (26–52) and in the presence of excess BAFF the median (range) CTI was 50 (12–75). However, even in this small number of samples the CTI of obinutuzumab was significantly greater than rituximab regardless of excess BAFF (*P* < 0.05).

## Discussion

Here, we found that BCD in SLE was of shorter duration than in RA. We report a disparity between peripheral blood BCD and renal BCD in individual patients with LN. Although this study did not find any significant relationships between the possible contributing factors investigated and BCD, obinutuzumab was superior to rituximab at inducing BCD, *in vitro*, regardless of excess BAFF.

Despite treatment with the same dose of rituximab, the duration of BCD was remarkably variable between individual patients with RA and SLE, as reported previously [1–3, 32–34]. Before treatment with rituximab, patients with RA had higher CD19+ B cell counts compared with patients with SLE. However, the observation that CD19+ B cells were detectable in peripheral circulation at an earlier time point after treatment with rituximab in patients with SLE compared with RA suggests that BCD was less pronounced and/or that repopulation of distinct B cell subpopulations occurred sooner in patients with SLE compared with those with RA and/or that CD19+CD20– plasma blasts and plasma cells, which may, at least in part, explain the disparity in clinical response reported in clinical trials involving patients with RA and SLE.

Despite disappointing trial evidence [4], rituximab is used especially in those patients who are refractory to standard treatment [35]. In LN, recruitment of macrophages, T cells and B cells into the interstitium contributes to inflammation [36], and in Class IV LN both activity and chronicity correlate with B cell infiltration in the interstitium rather than in the glomerulus [37]. Our data indicate a clinically relevant discrepancy in BCD between peripheral blood and the kidney in patients with LN with the persistence of interstitial B cells (as demonstrated by CD79- and Pax-5-positive cells), which was associated with progressive CKD and end-stage renal failure in four patients, whereas the patient with preserved renal function had no detectable B cells in the kidney. The only patient without any detectable B cells by either marker (CD79 or Pax-5) had a Class V lesion and at long-term follow-up has normal kidney function and remains in remission. Despite undetectable peripheral CD19+ B cells, the persistence of B cells following rituximab was associated with risk of progressive CKD and end-stage renal failure. Taken together with the observations that rituximab treatment leads to resorption of immune deposits in responding patients with LN [38], our findings suggest that improving BCD in the kidney may enhance clinical response to BCD therapy in LN.

We have previously reported that BCD in SLE is less efficient regardless of serum rituximab levels [14], which suggested that alternative resistance mechanisms operate to reduce the efficiency of rituximab. Although the study was not primarily designed to evaluate the effect of each parameter, we found no significant correlation between the CTI of rituximab and/or obinutuzumab and patient’s age, duration of disease or serum C3 levels. In contrast to some B cell malignancies, B cell expression of FcγRIIb and/or CD20 did not correlate with the *in vitro* efficiency of mAbs in either RA or SLE, with respect to the target cell expression of FcγRIIb [28, 39] and CD20 [40, 41]. Thus, although B cell expression of CD20 and/or FcγRIIb was variable between individuals and also in B cell subpopulations, the findings described here suggest that the thresholds of B cell expression of CD20 and FcγRIIb and/or baseline composition of B cell subpopulations do not seem to influence the efficiency of anti-CD20 mAbs in inducing BCD, *in vitro*, in RA and SLE patient samples.

There were no remarkable differences in susceptibility of B cell subpopulations to anti-CD20 mAbs in whole blood BCD assays. Allowing for the limitation that alterations in one B cell compartment would alter the frequency of other B cell subpopulations, the results suggest that IgD-CD27-B cells might resist depletion with rituximab, but not obinutuzumab in *in vitro* whole blood BCD assays.

BAFF inhibits apoptosis in B cells by up-regulating the anti-apoptotic factors Bcl-2 and Bcl-XL [42]. Therefore, BAFF may antagonize rituximab-mediated apoptotic cell death whereas BAFF may have limited effect on obinutuzumab-induced lysosome-mediated direct cell death [10, 18]. While the results of trials investigating the efficacy of combination therapy with rituximab and belimumab are awaited, a recent mechanistic study of the combination therapy in 15 patients followed up for 2 years reported that poor BCD was more common in non-responders than in responders [43]. Furthermore, patients who do not respond to rituximab due to incomplete depletion may benefit from treatment with an alternative anti-CD20 agent rather than belimumab [8]. Here, we found no consistent effects of BAFF on the efficiency of mAb-mediated BCD in this small number of samples. However, the efficiency of mAbs was not reduced in the presence of excess BAFF, whereas obinutuzumab induced superior BCD to rituximab regardless of additional BAFF in whole blood assays.

The main limitations of this study include that experiments to investigate the mechanisms behind the variability in BCD as well as comparing the efficiency of Type I and II anti-CD20 mAbs were carried out *in vitro*. In contrast to randomized controlled trials of rituximab in LN [4, 44], which failed to demonstrate superiority over standard of care, a phase II study demonstrated efficient peripheral BCD and renal responses with obinutuzumab compared with placebo when added to MMF and steroids [24]. Our data correspond with this in that obinutuzumab was more efficient than rituximab at inducing BCD in whole blood assays.

Thus, the main conclusions are: (i) B cell depletion in SLE is less efficient than RA; (ii) renal B cell infiltration following rituximab treatment is associated with poor outcomes; (iii) no significant relationships between the measured B cell intrinsic and extrinsic factors and the efficiency of BCD by rituximab; and (iv) obinutuzumab was superior to rituximab at inducing BCD *in vitro*, regardless of excess BAFF.

Taken together, this study highlights the potential of obinutuzumab, a commercially available, mechanistically different Type II anti-CD20 mAb with an afucosylated Fc portion not disposed to internalization (unlike rituximab), as an alternative B cell depleting agent in RA and SLE.

## Supplementary Material

keab827_Supplementary_Data

## Data Availability

The data are available from the corresponding author.
