# Supplementary material for: Disparity in peripheral and renal B-cell depletion with rituximab in systemic lupus erythematosus: an opportunity for obinutuzumab?
Source: Rheumatology (Oxford). 2021 Nov 11;61(7):2894–904. doi: 10.1093/rheumatology/keab827 (PMC9258539; doi:10.1093/rheumatology/keab827)
Supplement: keab827_Supplementary_Data [file keab827_supplementary_data.docx]

**Supplemental data**

Flow cytometry analysis of samples from patients with rheumatoid arthritis and systemic lupus erythematosus.

**Figure S1. Flow cytometry gating strategy to analyse the mean fluorescence intensity of CD20 and FcγRIIb (CD32).**

**Figure S2. Whole blood B-cell depletion assay.** Flow cytometry gating strategy for analysing anti-CD20 monoclonal antibody induced B cell cytotoxicity in whole blood assays.
